# The Etiology and Clinical Evaluations of Neonatal Seizures in Kashan, IRAN

**Published:** 2015

**Authors:** Ahmad TALEBIAN, Mohammad JAHANGIRI, Mahin RABIEE, Negin MASOUDI ALAVI, Hossein AKBARI, Zohreh SADAT

**Affiliations:** 1 Pediatric Department, Kashan University of Medical Sciences, Kashan, Iran; 2 Trauma Nursing Research Center, Medical Surgical Department, Kashan University of Medical Sciences, Kashan, Iran; 3 Department of Statistics, Kashan University of Medical Sciences, Kashan, Iran; 4 Trauma Nursing Research Center, Midwifery Department,Kashan University of Medical Sciences, Kashan, Iran

**Keywords:** Etiology, Seizures, Neonate, Kashan

## Abstract

**Objective:**

Detection of seizure, its etiology, and clinical types is important for guiding therapy. This study was designed to evaluate the etiology and clinical evaluations of neonatal seizures in Kashan, Iran.

**Materials and Methods:**

The data of 100 hospitalized neonates with a complaint of seizures in Kashan City, from January 2006 to January 2011 were evaluated. The pediatric neurologist made the final diagnosis. The gestational age, neonate admission age, type of delivery, and laboratory and radiological investigations were reviewed from the medical records. The relation of seizure etiology and other variables were compared using the Chi-square test. All the statistical analyses were performed using SPSS (ver 11.5).

**Results:**

A total of 100 neonates were hospitalized with a diagnosis of seizures. The overall incidence rate of seizures was 2.6 per 1,000 live births. A total of 59% of seizures happened in the first three days of life. The etiologies of seizures were hypoxicischemic encephalopathy (HIE) (36%), hyponatremia (12%), hypoglycemia (11%), intracranial hemorrhage (11%), infections (10%), hypocalcemia (8%), metabolic disorders (7%), the structural anomalies (5%), and hypomagnesaemia (4%). In 23% of neonates, no specific etiology was found and 23% had multiple etiologies. In 45% of neonates, the EEG was not recorded. The type of the seizures were focal-clonic (26%), tonic (25%), multifocal clonic (34%), subtle (11%), and myoclonic (4%). The types of the seizure were unrelated to the paraclinical findings.

**Conclusion:**

Neonatal seizures are common and HIE was the main cause of seizures in this study. The clinical evaluation of neonatal seizures needs improvement.

## Introduction

Seizures are a common and the most frequent neurological presentation in neonates (1). They are a major risk of death and long-term morbidity including mental retardation in neonates (2, 3). The incidence of seizures varies widely in different countries. The incidence has been reported from 1.8–5 per 1,000 live births in the United States of America (1) to 39.5 per 1,000 live births in Kenya (4). The incidence is higher in premature and low birth weight infants (2, 5, 6). Current estimates indicate the prevalence of neonatal seizures ranges from 0.5% in full-term to 22.2% in preterm newborns (2). Birth asphyxia (7), congenital brain anomalies, and sepsis are common etiologies of neonatal seizures (8). Hypoxicischemic encephalopathy (HIE), affects approximately 1–2 per 1,000 live births. These seizures occur following birth asphyxia and respiratory distress. Infection is also a common cause of neonatal seizures. Common bacterial infectious causes are Group B streptococcus and Escherichia coli. Malformations of cortical development and metabolic disturbances including hypoglycemia, hypocalcemia, and hypomagnesaemia are other causes of neonatal seizures (5). In Kenya, the main diagnoses of neonate seizures were sepsis and neonatal encephalopathy. The higher rates of neonatal seizures in developing countries may be because of higher neonatal sepsis (1). A clinical diagnosis of seizures is a challenging issue. One study revealed that 80% of electroencephalography (EEG) documented seizures were not accompanied by observable clinical seizures (4). In another study, only 27% of clinical seizures were correctly identified, and 73% of presumed clinical seizures had no electrographic correlate, which led to over diagnosis (9). Hence, EEGs are considered essential for the diagnosis of seizures in neonates (4, 10). Once neonatal seizures are confirmed, treatable metabolic and symptomatic causes need to be identified. Serologic studies, metabolic evaluations, MRIs, and ultrasonography are important in the assessment of neonatal seizures. Imaging can provide information of gross structural malformations (4, 11). In a study in Iran, neonatal seizure was reported in 3.6% of neonates. The most frequent etiology was neonatal sepsis (8). Although neonatal seizures are a common problem in hospitalized neonates, few Iranian studies have examined the causes of seizures. Detection of seizure, its etiology, and clinical type is important for guiding therapy and helping to determine the risks of morbidity and mortality. This study was designed to evaluate the etiology and clinical evaluations of neonatal seizures in Kashan, Iran. 

## Materials & Methods

The data from all neonates who were hospitalized with a complaint of seizures from January 2006 to January 2011 in the Shahid Beheshti Hospital in Kashan city were evaluated. The hospital is located within the Isfahan Province in the center of Iran and it is the only general hospital in the region with a population of 400,000. In the existent data, a cross sectional study where a neonate was defined as any child admitted aged 28 days or younger (12). Seizures were defined as repeated involuntary muscle contractions, abnormal tonic extensions, or jerky movements of any part of the limb, face, or mouth that were not stimulus sensitive or repetitive abnormal chewing, ocular, or pedaling movements (4). The pediatric neurologist made the final diagnosis for all neonates with seizures. The gestational age, neonate admission age, type of delivery, and laboratory and radiological investigations were reviewed from the medical records. HIE was diagnosed according to the clinical data, laboratory findings, and imaging findings. The infection diagnosis was made upon isolation of pathogenic organisms from blood or cerebrospinal fluid (CSF) cultures. Prematurity was considered in any neonate born before 37 completed weeks. All blood samples were analyzed in the same laboratory for blood glucose, electrolytes, and microbiological culture. Hypokalemia was defined as plasma K < 3.5 mEq/L; Hyperkalemia as K> 7 mEq/L, hyponatremia (moderate-severe) as Na< 45 mg/dL; hypocalcemia as Ca< 1.5 mg/dL. The total population of live births in the demographic surveillance area at the mid-point of the study was estimated in order to calculate the incidence of neonatal seizures. The rates are expressed as events per 1,000 live births per year. The relation of seizure etiology and other variables were compared using the Chi-square test. All the statistical analyses were performed using SPSS (ver 11.5). Ethical considerations: The study was approved by Kashan University of Medical Sciences ethical committee. All personal information was kept anonymous during the study.

**Table 1:** The Relation of Seizure Etiologies and Other Variables

Multiple Causes	Infections	Hypomagnesaemia	Hyponatremia	Hypocalcemia	Metabolic Disease	Structural anomalies	Intracranial Hemorrhage	Hypoglycemia	HIE		
13 (31)	5 (11.9)	2 (4.8)	5 (11.9)	5 (11.9)	4 (9.5)	4 (9.5)	1 (2.4)	6 (14.2)	16 (23.8)	CS	Delivery N (%)
10 (17.2)	5 (8.6)	2 (3.4)	7 (12.1)	3 (5.2)	3 (5.2)	1 (1.7)	10 (17.2)	5 (8.6)	20 (34.5)	NVD	
NS	NS	NS	NS	NS	NS	NS	0.019	NS	NS	P value	
6 (46.2)	3 (23.1)	1 (7.7)	3 (23.1)	0	1 (7.7)	1 (7.7)	1 (7.7)	1 (7.7)	9 (69.2)	Preterm	Gestational age N (%)
17 (19.5)	7 (8)	3 (3.4)	9 (10.3)	8 (9.2)	6 (6.9)	4 (6.6)	10 (11.5)	10(11.5)	27 (38)	Term	
0.033	0.012	NS	NS	NS	NS	NS	NS	NS	0.007	P value	
18 (27.7)	6 (9.2)	3 (14.6)	5 (7.7)	4 (6.2)	6 (9.2)	4 (6.3)	8 (12.3)	9 (13.8)	30 (46.2)	7days ≥	The age of infant N (%)
5 (14.3)	4 (11.4)	1 (2.9)	7 (20)	4 (11.4)	1 (2.9)	1 (2.9)	3 (8.6)	2 (5.7)	6 (17.1)	7days<	
NS	NS	NS	NS	NS	NS	NS	NS	NS	0.004	P value	
13 (28.3)	5 (10.9)	4 (8.7)	4 (8.7)	4 (8.7)	5 (10.9)	2 (4.3)	5 (10.9)	6 (13)	17 (37)	Male	Sex N (%)
10 (18.5)	5 (9.3)	0	8 (14.8)	4 (7.4)	2 (3.7)	3 (5.6)	6 (11.1)	5 (9.3)	19 (35.2)	Female	
NS	NS	0.027	NS	NS	NS	NS	NS	NS	NS	P value	

NS= Non-significant

**Table 2:** The Paraclinical Findings in Neonates with Seizure

**Paraclinical findings**	**Normal**	**Abnormal**	**Not evaluated**
Hypoglycemia	89	11	0
Hypocalcemia	90	8	2
Hyponatremia	86	12	2
Hypomagnesaemia	71	4	25
Cerebral spinal fluid culture	36	10	54
Sonography	61	12	27
EEG	17	38	45
CT/MRI	14	26	60
VBG	55	42	3

**Fig 1:**
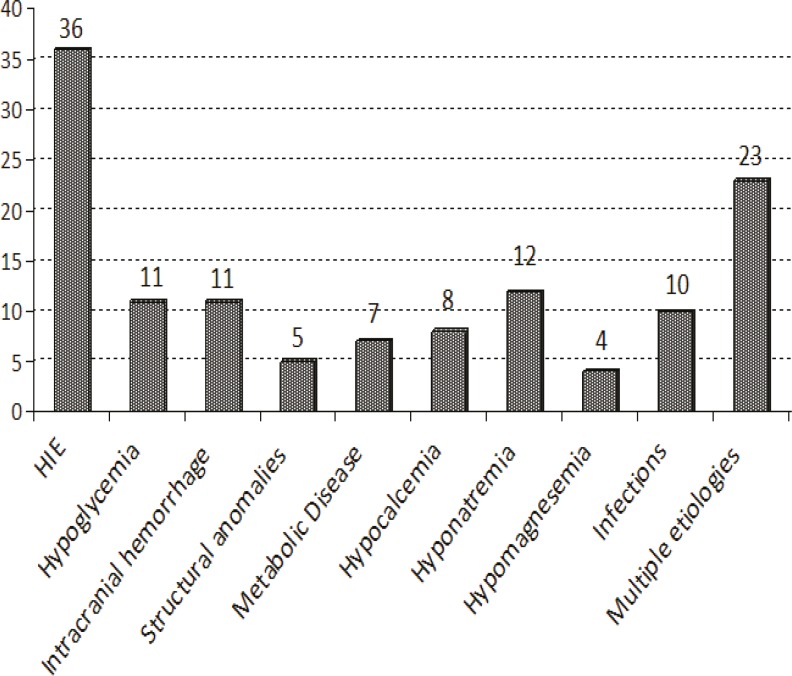
The etiology of seizures in neonates

## Results

From January 2006 to January 2011, 100 neonates with a final diagnosis of seizure were hospitalized in the Shahid Beheshti Hospital in Kashan City. The overall incidence rate of seizures was 2.6 per 1,000 live births per year. All seizures occurred between the first and 28th days after birth. A total of 59% of neonates had seizures in the first three days of life, with the mean age of 8.2 ± 9.4 days. A total of 46 neonates were female and 54 were male with 16% of parents had familial consanguinity. A total 87% of neonates were term and 13% were preterm. The etiologies of seizures were HIE (36%), hyponatremia (12%), hypoglycemia (11%), intracranial hemorrhage (11%), infections (10%), hypocalcemia (8%), metabolic disorders (7%), the structural anomalies (5%), and hypomagnesaemia (4%). In 23% of neonates, no specific etiology was found and 23% had multiple etiologies, That is the reason why the over all etiology are more than 100% (Figure-1). A total of 10 neonates had a diagnosis of infection, 2 neonates had TORCH infections (1 toxoplasmosis and 1 cytomegalovirus infection), 4 had meningitis, 2 had pneumonia and 2 had urinary tract infections. Table 1 presents the relation between seizure etiology and other variables. Intracranial hemorrhage as an etiology of seizure was significantly higher (p-value= 0.019) in normal vaginal delivery (NVD) compare to cesarean section (CS). HIE and infection were significantly higher in preterm neonates (p-value = 0.007). HIE as a cause of seizure was significantly more in the first week of life (p-value = 0.004). Hypomagnesaemia was significantly more in male infants (p-value = 0.027). In paraclinical findings, the Venus blood gas (VBG) and EEG were abnormal in 42% and 38% of neonates, respectively. The 26% of neonates had abnormal neuroimaging (CT, Sonography, or MRI). Hyponatremia (12%), hypoglycemia (11%), and hypocalcemia (8%) were the most common biochemical findings. Hypomagnesaemia was found in 4% of neonate. A total of 10% of neonates had positive and 36% had negative CSF or blood culture; while 54%, the CSF or blood cultures were not evaluated. In 45% of neonates, the EEG was not recorded. Table 2 presents the paraclinical findings. The type of the seizures were focal-clonic (26%), tonic (25%), multifocal clonic (34%), subtle (11%), and myoclonic (4%). The types of the seizures were unrelated to the paraclinical findings.

## Discussion

In this study, the overall incidence of seizures was 2.6 per 1,000 live births per year. The exact incidence of neonatal seizures in the general newborn population is difficult to estimate. The incidence of neonatal seizures has been estimated using clinical observation of abnormal movements. The clinical presentation of seizures during the neonatal period is subjective, leading to considerable variability in their recognition and diagnosis (2). The incidence of seizures in current study was comparable with some other studies (2, 5); although it is much lower than incidences reported in Kenya (1) and Ethiopia (13). The reason might be that sepsis is lower in Iran or because in this study only the charts of hospitalized neonates were evaluated. Thus, our data could underestimate the seizures in the neonates. A previous study showed that only 20% of children with seizures might have been admitted to the hospital (1). The main etiology of seizures was HIE (36%). In a review, HIE was responsible of 40–50% of neonatal seizures (2), another study also showed that the 53.9% of neonatal seizures had occurred following HIE (14). Yildiz et al. also found that asphyxia (28.6%) is the main cause of neonatal seizures in Turkey (15), which is comparable to our study. In one study, hypoglycemia was reported in just 0.1–5% of neonatal seizures (2), which was lower than 11% that has been reported in our study. Intracranial infections are the most common cause of seizures in developing countries (8, 1). In one review, the 6–17% of seizures was due to intracranial infections (14). In our study, 10% of seizures were due to infections and, although, it was not the main etiology of the seizures. In Hormozgan, Iran, the infection has been the etiology of seizures in 19.1% of neonates, which is higher compared to the current study (16). In Loman et al Study only 0.5% of neonatal seizures had unknown etiologies (14). Yildiz also did not find any etiology in 8.9% of neonates (15). The unknown etiologies were much higher in our study, which might show the clinical and paraclinical investigations were not adequate. Some studies show that gender is unrelated to neonatal seizures, although it was otherwise identified as a higher risk for male infants (2). We also did not find a relation between gender and the occurrence of seizures. A third of the seizures occur in the first day and another third within the first week of life (2). In our study, also 59% of seizures occurred in the first three days of life. The etiology of HIE was also significantly higher in seizures of the first 7 days of life. EEG recording is essential for accurate identification of neonatal seizures (17, 18, 19). Conventional electroencephalography (cEEG) is the standard method for diagnosis of neonatal seizures (20). In another study, the methods of diagnosis of seizures were studied. EEG was the most commonly used modality (58%), although 33% used either amplitude-EEG or EEG, and 8% accepted clinical observation alone for the diagnosis of seizures. Head ultrasound (HUS) and magnetic resonance imaging (MRI) were commonly used (more than 75%) in term and preterm infants (21). Ultrasound offers a rapid tool with the possibility of identifying intracranial pathology. A cranial ultrasound is routine management in the investigation of neonatal seizures. Magnetic resonance imaging (MRI) is the ‘gold standard’ in the examination of the newborn brain and will reveal most brain pathology (18). In our study, the EEG was recorded in only 55% of neonates and CT scan and MRI were done in 40% of cases, which was less than recommendations. The 73% of neonates had head sonography, which is comparable to Glass (21). In our study, all the neonates were evaluated for hypoglycemia. Hypoglycemia is a common cause of neonatal seizures that can be rapidly corrected so its diagnosis is important (18). The CSF culture was performed in 46% of neonates. Any infectious suspicion warrants a laboratory work-up including lumbar puncture for culture (18). The type of seizures and its prognostic value remain a challenge for the clinician, a number of studies report that subtle seizures have a worse outcome compared with those with clonic seizures (11). In our study, only 11% of seizures were subtle and most neonates had tonic-clonic seizures, which is comparable with the literature (1).

**In conclusion**, the incidence and etiologies of the neonatal seizures in the current study were comparable with developed countries, which might reflect the acceptable control of neonatal infections in our country. Although, 23 of neonatal seizures had no specific etiology and only 55% had EEG in their medical records, the diagnosis of neonatal seizures might not be according to standards . In this study, we only evaluated the medical records of hospitalized infants, which is a limitation of this study. It is possible that we have missed subclinical seizures. There is also a risk of underestimating neonatal seizures in this study. We recommend the prospective design for future studies. The findings show that existent data in Kashan City, so there must be precaution in the generalization of our results. We did not follow neonates for short- or long-term outcomes. Future studies may address this important issue.

## Notes

**Funding Support:** This research has been supported by Kashan University of Medical Sciences.
